# Notes and News

**Published:** 1902-03-01

**Authors:** 


### Notes and News.

HOSPITAL MEETINGS.

Royal Hospital for Children and Women.—Annual Meeting, Monday, March 3rd, at 3 P.M., at Mansion House.

Mount Vernon Hospital for Consumption.—Annual Meeting, Wednesday, March 5th, at 4 P.M., at 41 Fitzroy Square.

London Hospital.—Quarterly Court, Wednesday, March 5th, at 3.30 P.M.

West Ham Hospital.—The Annual General Meeting will be held under the presidency of Thomas Cook, Esq., at the Dispensary Hall, West Ham Lane, on March 5th, at 8 P.M.

New Hospital for Women.—Annual Meeting, Thursday, March 6th, at 3 P.M.

Royal Westminster Ophthalmic Hospital.—Annual Meeting, Friday, March 7th, at 4.30 P.M.

The London Temperance Hospital.—The Annual Meeting will be held at the Hospital on March 24th, at 7 P.M.

---

The King's expression of interest in the subject of cancer will, no doubt, cause even a larger amount of attention to be drawn to it publicly than the suggested Commission. Royal interest in any cause is always of immense practical value, and generosity always follows in its footsteps. Tuberculosis has already benefited greatly, and it is to be hoped that the more terrible disease will be fought with greater prospect of success, through large donations, more especially in research, as in cancer there is ample room for a very large expenditure.

---

The Midwives Bill passed the second reading in the House of Commons on Wednesday without division, and is referred to the Standing Committee on Law.

---

Glasgow is again unfortunately tainted with small-pox. Upwards of 130 persons are under treatment. Most of the cases have occurred in model lodging-houses. The recent epidemic was not sufficient to teach the inhabitants a lesson, and the number of persons unrevaccinated is large to-day.

---

It is somewhat strange that the recent order excluding tuberculous persons from entering the United States has not met with approval from the medical profession in that country generally. They approve of the exclusion of the alien pauper suffering from tuberculosis, but regard the regulation as hard upon other aliens affected by the disease, which they do not regard as dangerously infectious if due precautions are taken.

---

A public memorial is to be raised to the late Surgeon-General R. Harvey, C.B., Director-General of the Indian Medical Service.

---

The Right Hon. Lord Monkswell will preside at the 63rd Anniversary Dinner in aid of the Newsvendors' Benevolent and Provident Institution on Wednesday, May 7th.

---

The German Congress of Internal Medicine will be held this year at Wiesbaden, commencing on April 15th, under the presidency of Professor Naunyn. The programme is long and comprehensive.

---

Lord Lister, Professors Virchow, Koch, Lombroso, Golgi, Retzius, Politzer, Cajal, Rodriguez, and Mendez y Carricido have been invited to deliver addresses at the International Medical Congress at Madrid in 1903.

---

The conscientious objector is certainly not gaining ground. In 1898 the vaccination officers recorded 203,417 conscientious objections, and in 1900 only 39,839. The number of vaccinations shows a decided increase during the same period.

---

Medical men who desire to combine the pursuit of pleasure, health, and knowledge have very good and varied opportunities offered them by the various medical congresses to be held this year. In September, the German Congress of Medical Scientists and Practitioners meets at Carlsbad, and in December the first Egyptian Medical Congress will be held in Cairo. The use of any language will be permissible at the last, though the official languages will be French and Arabic. The French Medical Congress will be held at Toulouse on April 1st.

---

To those who have studied the various medical publications issuing from the medical centres of the many states of America, it must always have been a matter of wonder, how it was possible for such varying regulations to exist under one Government, and for the medical practitioners to work with reasonable facility under the many and various jurisdictions. With the object of removing these difficulties a Federation of Medical Boards is being organised. The federation will devote its energies to securing uniformity and co-operation between the medical societies everywhere.

---

Dr. C. Ball, member of the Advisory Board to the Army Medical Service, in his recent address at Dublin pointed out several factors which were responsible for the dearth of candidates for the Army Medical Service. He showed that young medical men of the present time have a better prospect in civil practice. The abolition of the unqualified assistant had opened a larger field to qualified medical men, whilst the supply had been decreased temporarily by the lengthened curriculum now required and which delayed the students of one year from being available. This lengthened course of study renders the production of a medical man to be more costly, and therefore increased remuneration was looked for.

